# Two Physiotherapy Methods to Improve the Physical Condition of Children with Autism Spectrum Disorder

**DOI:** 10.3390/children11070798

**Published:** 2024-06-28

**Authors:** Lina Draudvilienė, Justas Draudvila, Simona Stankevičiūtė, Laura Daniusevičiūtė-Brazaitė

**Affiliations:** 1 Ultrasound Research Institute, Kaunas University of Technology, LT-51423 Kaunas, Lithuania; 2 Department of Applied Biology and Rehabilitation, Lithuanian Sports University, LT-44221 Kaunas, Lithuania; juokas2@gmail.com; 3 Medical Academy, Lithuanian University of Health Sciences, LT-44307 Kaunas, Lithuania; simona.stankeviciutee@gmail.com; 4 Faculty of Social Science, Arts and Humanities, Kaunas University of Technology, LT-51424 Kaunas, Lithuania; laura.daniuseviciute@ktu.lt

**Keywords:** autism spectrum disorder, physical therapy session, smart board games, gym, balance, coordination, motor skill

## Abstract

This study presents two simple physiotherapy programs that were implemented for five weeks and showed positive changes in balance, coordination, and motor skills in kindergarteners with ASD. Physiotherapy programs in a gym and games on a smart board with balance plates and an unstable base were applied to improve the physical condition of children with ASD. Thirty children with ASD (4–6 years old) attending special needs kindergarten were enrolled in the study. Three tests were used to assess participants’ physical condition before and after the study: the modified Berg Balance Scale, the Imbalance Coordination Sample, and the Bruininks–Oseretsky Motor Proficiency Test (BOTMP). The resulting mean change, calculated from each group’s scores, shows that the participants who received physical therapy sessions at the smart board had the greatest change of 1.58 points. It shows that the opportunity to play games on a smart board motivates children with ASD to work harder; therefore, it is a simple and easy way to engage children in different types of physical exercise. A slightly smaller change of 1.51 was obtained in the group that received gym sessions. However, working in the gym was more psychologically challenging for the children with ASD due to their lack of desire and motivation. Both methods are relatively simple and easy to apply at home; therefore, parents can make a significant contribution to improving children’s physical condition and that can be an effective tool to assist these individuals with activities in daily life.

## 1. Introduction

Autism disorder is mainly characterised by core features in two areas: social communication and motor behaviour with restricted, repetitive sensory behaviour [1]. At the physical level, it can manifest as impaired coordination and balance and could result in postural stability challenges and increase the risk of injury [2,3,4]. However, several individuals with ASD struggle with postural stability [5]. These postural stability difficulties have been linked to autism symptoms and have been shown to persist into adulthood and are marked by an atypically early plateau during adolescence [6].

A meta-analysis review of 15 previous studies focusing on balance measures showed that any type of physical intervention programme is beneficial for children with ASD [7]. Children can improve their motor skills, balance, and fitness levels by participating in different PA intervention programmes [3,7,8,9,10]. It was found that PA has a positive impact and can be an effective tool to assist these individuals with activities in daily life that require strength, endurance, flexibility, and stability [11] and can help to increase the self-confidence of children with ASD [3,7,12].

Longer-term changes in postural stability in ASD have additionally been reported from a 12-week hippotherapy intervention [13] and a 6-week balance–exercise training program (single-leg stance, double-leg stand, and walking along designated paths under different sensory conditions) [14]. These studies offer preliminary evidence that balance can be improved in individuals with ASD; however, adherence to long-term training may be difficult unless the training can be implemented in a fun and motivating way.

Therefore, increasingly, different ways are being sought to improve these children’s functional abilities, physical stability and activity that would facilitate their integration into society and the psychological quality of life [15]. However, due to their atypical behaviour and social isolation, they lack the skills to engage in PA [16,17]. Other barriers to successful participation in PA include limited resources and staff training, and parents may lack information about available PA programmes and training on how to do them [16,18]. Lack of motivation could also be named as one of the main reasons why children with ASD do not participate in PA. Therefore, children with autism do not exercise and as a result, they have poorer physical health and are less physically fit than typically developing children [19,20,21]. A study of children up to 16 years of age found that children with ASD were PA for only 31 min on weekdays, compared to 55.2 min for typically developing children [15]. Therefore, to improve the physical condition of these children, it is necessary to find the best way to include such children in PA [11].

With the rapid advancement of technology, various tools are being developed that can encourage the development of children with ASD and their physical activity [22]. Children with ASD have been found to play computer games longer than children without the disorder [23]. Boys with ASD spend an average of 2.4 h playing computer games and girls spend 1.8 h per day, while typically developing children spend 1.6 and 0.8 h, respectively [24]. Thus, computer games are one of the interests of children with ASD. However, it has been observed that children are not physically active while playing computer games, so this has a negative effect on the physical characteristics of these children and increases the likelihood of obesity [24]. As a result, various types of equipment such as the Wii, virtual glasses, Xbox Kinect, and smart boards have recently been developed to allow children to play computer games while engaging in physical activity [24]. Using this equipment, computer games are controlled by the movements of the players as they stand, copy the displayed movements or move in a small space. Such games encourage children to move and are a useful way to activate and educate children [25,26]. Thus, this type of training is a fun and motivating method of targeting physical condition challenges in individuals with ASD.

The main idea of this work is to propose two physiotherapy methods, namely a physiotherapy programme in a gym and games on a smart board with balance plates and an unstable base, to improve the physical condition of children with ASD; to evaluate which method is more effective in improving balance, coordination, and motor skills; and to present which method motivates children more for physical activity.

Based on a literature review, it is hypothesized that if a certain exercise program in the gym and at the smart board is applied consistently for 5 weeks to children with autism spectrum disorder, PT will help improve their balance, coordination, and motor skills.

## 2. Materials and Methods

### 2.1. Participants

**Figure 1:**
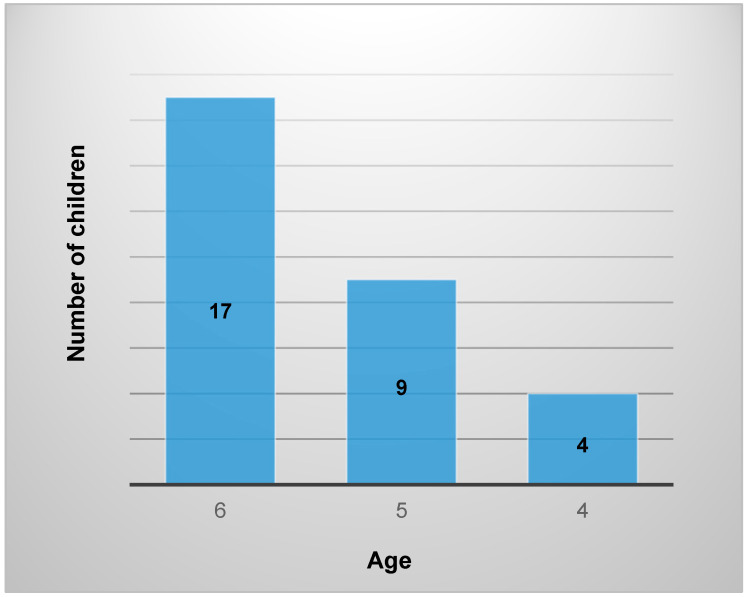
Distribution of children by age.

Thirty children—six girls and twenty-four boys—attending a special needs kindergarten participated in the study. The inclusion criterion was a clinical diagnosis of ASD. All participants with ASD had code F 84.0 in their cases and fulfilled the criteria for ASD according to the Diagnostic and Statistician Manual of Mental Disorders (DSM-V) [American Psychiatric Association, 2014] [27]. The distribution of participants by age is shown in Figure 1.

Aggressive behaviour, intellectual disability, or attention deficit hyperactivity disorder (ADHD) were not identified. All participants were of a similar developmental level, therefore significant differences between children of 4 and 6 years of age were not determined. The physical characteristics of the children are: height 114.77 cm ± 9.05 and weight 21.55 kg ± 6.73. The age of the participants was 5.37 years ± 0.76.

Family members were asked not to start any new exercises or physiotherapy programmes for the participants during the 5 weeks of training, and to ensure that the children attend all physiotherapy sessions.

### 2.2. Study Design and Procedure

**Figure 2:**
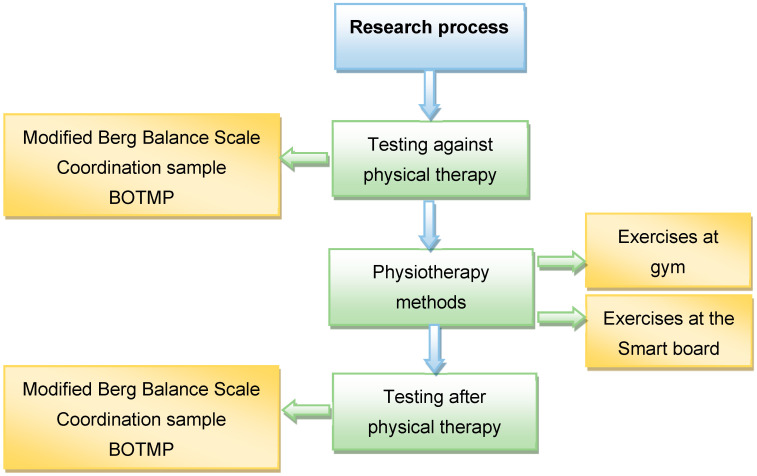
The algorithm of the research process applied individually to each participant.

The research carried out consisted of three stages: pre-test, intervention, and post-test. In the first phase, the children were tested using three selected methods that determine balance, coordination and motor skills. In the second phase, two selected physiotherapy methods were applied: gym exercises and games on a smart board, which could be accessed by crossing an unstable base. In the third phase, the children were retested using the same testing methods. The algorithm of the research process, applied individually to each participant, is shown in Figure 2.

Firstly, the children were tested for balance. The first test chosen was the pediatric balance scale (modified Berg balance scale). This test was chosen because it is suitable for use in preschool children with known balance problems [28]. It is a functional skills assessment method for balance assessment, which consists of 14 tasks, and the total time for these tasks is 20 min. With the help of this test, the functional abilities required in daily activities related to balance are determined. Balance is established by changing position and performing various movements simultaneously. The tasks are performed while sitting, standing, and with eyes closed. The modified Berg Balance Scale consists of a 5-point system from 0 to 4 (0–1–2–3–4). The maximum number of points that can be collected is 56 points. If the final result of the test is less than 46 points, it is considered that the person has serious balance disorders, which increases the probability of falls [29].

Secondly, the children’s coordination was tested using the non-equilibrium coordination sample (Schmitz, 1988) [30]. Also, during these samples, the large and small muscles are included, providing a broader understanding of the research area. All coordination samples describe five basic movement possibilities: (1) alternative or counter movement; (2) movement composition or synergy; (3) accuracy of movements; (4) maintaining a limb or part of it in a certain position; (5) maintaining static and dynamic balance while standing (Schmitz, 1988) [30]. This coordination test consists of 15 tasks and each task is scored from 0 to 4 points. Points are assigned according to the test regulations: 0 points—accurate movements not performed, body position not maintained; 1 point—it is very difficult to maintain the body position, the movements are arrhythmic, imprecise, tremors appear, side movements; 2 points—maintaining the body position is a struggle, the movements are irregular and become even more imprecise when the performance speed is increased; 3 points—movements and body position are difficult to perform and maintain, inaccuracies occur; 4 points—normal static and dynamic body position was maintained, movements were performed in accordance with all requirements. The maximum test score is 60 points.

Thirdly, the children’s motor skills were tested. The test was chosen because it has been already applied with positive results obtained for preschool children in Lithuania [31]. Therefore, the methodology of the Bruininks–Oseretsky Test of Motor Proficiency (BOTMP) is suitable for assessing the motor skills of preschool children. This test is divided into three parts: gross motor skills, general motor skills, and fine motor skills. The overall course of the study and the analysis of the results obtained are presented in the following sections.

#### Physiotherapy Procedure and Methods

Two Physiotherapy Programmes (PP) are used for the study, one consisting of a simple exercise programme in a gym and the other consisting of games on a smart board with balance boards and an unstable base. Both PPs were applied in the children’s natural environment, an indoor gym. One of the researchers had already worked with autistic children for more than 3 years in the kindergarten. Some of the children participating in the study had known this person for at least one year. This person participated in all research stages. An additional person participated in the pre-test and post-test phases. Therefore, before starting the procedural tests, each child was communicated with for about one week. This allowed the children to get used to the new person and facilitated the assessment of each child’s perceptual abilities (listening and task performance). Then, each child’s primary condition was determined and assessed using the selected tests.

Before the study started, the children were first divided into age groups. Children from each group were then randomly selected and assigned to three different groups. Two groups represented a different application of the PP, and the third group was a control group. The children in the control group were involved in normal daily activities, with no additional physical activity.

- Physical activities in the gym. The chosen PP in a gym consists of simple exercises such as exercises on unstable surfaces, exercises with balls, throwing, catching, and hitting, as well as walking over obstacles to improve coordination. The kinesiotherapy programme used in the gym is shown in Table 1.
- Smart board games. Another selected PP to promote activity in children with autism is computer games. The smart board exercises were organized in such a way that the children had to reach the smart board by walking on an unstable surface to play on the board, and any touch or hand movement to the smart board was done while standing on the balance plates. The children played games on these websites: http://www.ziburelis.lt/ or http://www.frepy.eu/part_lt.html. (accessed on 6 November 2019).

The physiotherapy sessions took place 3 times a week and the whole study lasted 5 weeks. At the end of the entire study period, the children were tested again using the same test methods to compare the results obtained before and after the physiotherapy sessions. The results are presented in the next section.

## 3. Results

MC Office Excel 2013 was used for statistical analysis of the results obtained, and statistical data analysis was performed using the IBM SPSS statistical software package, version 23.0. The normality hypothesis was tested using the Shapiro–Wilk test. The Student’s *t*-test was used to compare two groups of quantitative data. Paired Student’s *t*-test was used to compare two dependent samples (test results before and after physiotherapy). One-way ANOVA was used to calculate the change between groups. The Post Hoc test was used to check which groups differed. The data of the indicators evaluated are presented as arithmetic mean and standard deviation (±SD). The results obtained are presented in graphs with the error of the mean. The data were statistically significant if *p* < 0.05.

**Figure 3:**
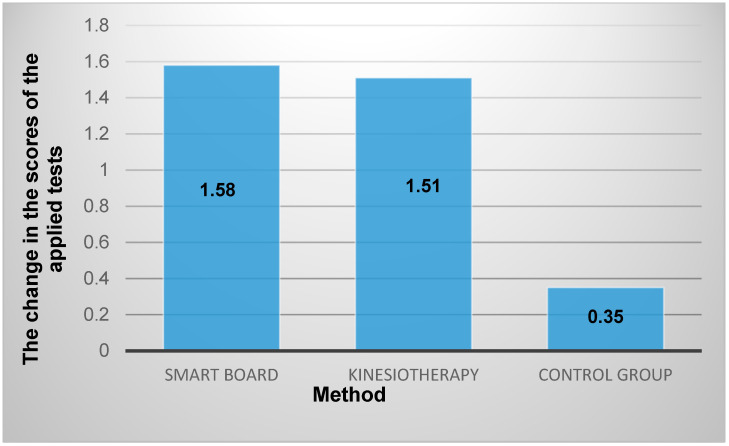
The change in the scores of the applied tests according to the obtained methods. *p* < 0.05 compared to control group.

The obtained data of the research conducted are presented in Table 2. The change in the results of the applied tests according to the obtained methods is presented in Figure 3. The discussion and comparison of the obtained results are given below.

The comparison of the obtained pre- and post-test results of the balance, coordination, and motor skills of the children with ASD who participated in the physiotherapy sessions (Table 2) shows that all the general results of the children improved after the use of both applied physiotherapy methods. Meanwhile, the results of the children in the control group did not change or changed very little.

## 4. Discussion

Two simple physiotherapy programs—the exercise program used in the gym and the additional exercises on the smart board that were implemented for five weeks—resulted in a positive change in balance, coordination, and motor skills in kindergarteners with ASD. The obtained results showed that consistent and constantly applied simple PAs can improve the physical condition and produce positive results for these children’s balance, coordination, and motor skills.

### 4.1. Results Achieved with Physiotherapy Exercises in the Gym

Comparing the results of children with ASD who participated in a PP in a gym (Table 2), the balance, coordination, and motor skills of these children improved significantly: the resulting mean change of 1.51 was detected (Figure 3). The results suggest that more robust training-related balance improvements in the game are generalized to better post-training postural stability. The results of balance improvement are consistent with other research, such as the use of the Biodex balance system or balance training programme [14,32]. Previous research has shown that repetitive behaviours are associated with postural stability in ASD [33]. The present findings introduce the possibility that more severe repetitive behaviours may impede improvements in balance training, which in part might account for the link between repetitive behaviours and balance in ASD. Alternatively, it is possible that co-occurring features in ASD, such as sensory features, may be driving these effects, as sensory features and repetitive behaviours have been found to be associated with ASD [33].

The results of the BOTMP coarse motor test show an improvement in the children’s running speed and agility, balance, bilateral coordination and strength (Table 2). The improvement in running speed and dexterity was mainly influenced by the tasks where the children had to collect balls in the hall as quickly as possible and put them in the basket. The improvement in balance has already been discussed. The improvement in bilateral coordination could be explained by the influence of an exercise such as walking and lifting the opposite hand to the leg, and other exercises in the gym: jumping through obstacles and others. These exercises may have helped to improve the strength results. The BOTMP general motor test showed a significant improvement in upper body coordination.

The first test of fine motor skills was the reaction speed test. During the gym sessions, a ball was thrown and the children had to catch it. The sessions significantly improved the reaction speed of the children with ASD. Visual motor control exercises were not used enough, so the results do not show a significant improvement. The last test used was the upper body speed and dexterity test and the results showed a significant improvement. This could have been influenced by the exercise of collecting balls as quickly as possible, throwing and catching the ball. The research carried out shows that exercises help children with ASD to improve their motor skills [8,34].

This PP is relatively simple and easy to do at home, so parents can make a significant contribution to improving their children’s physical condition.

### 4.2. Results Achieved with Smart Board Tasks

The resulting mean change, calculated from the scores of each group that attended the sessions, shows that the group that received PP on a smart board had the greatest change of 1.58 points (Figure 3). A study that evaluated children’s motivation in working with virtual reality found a high motivation and willingness to engage in such physical activities in children with ASD [35,36]. It was determined that video games can improve attentional control and sustained attention [37], reduce anxiety [38], improve daily life skills [39], and promote physical activity [40]. Activities with children showed that playing on the smart board also encouraged children’s willingness to engage in this activity. Therefore, exercises were organized in such a way that the children had to reach the smart board by walking on an unstable surface. Each time the children had to make another move at the smart board, they had to cross a specially constructed unstable base. Therefore, it can be said that this had an impact on the children’s balance improvement. The results obtained on balance improvement by playing the virtual games are also supported by other studies [25,41,42]. Since every touch or hand movement on the smart board was done while standing on the balance plates, it recruited and developed coordination. Our findings suggest that visual-based biofeedback training improves balance in ASD. These findings are consistent with previous research that postural stability in ASD can be improved through various means [25,43].

The results of the BOTMP gross motor test show an improvement in children’s balance and coordination. However, the results showing the children’s running speed, dexterity and strength did not show a significant improvement (Table 2). This is because the group of children who worked at the smart board were not given exercises to develop their running speed and dexterity.

The general motor test, BOTMP, showed a significant improvement in the children’s upper body coordination (Table 2), as the movements on the smart board are performed with the upper body. The last part of the BOTMP, the fine motor test, is a reaction and speed test that showed a significant improvement. This improvement is explained by the fact that the children played games that required finger motor skills. The visual motor control test also showed significant improvement. While playing on the smart board, the children had to distinguish figures, understand their shapes, and identify and classify them by colour; this is part of the visual motor tests. A final test that also showed significant improvement was upper body speed and dexterity. As some of the tests used were correlated with the games the children played on the smart board, the only difference is that the children had to do the tests on paper, whereas during the sessions the children solved the tasks on the smart board, which explains the significant improvement. Thus, play sessions using a smart board with balance boards and an unstable base help improve the motor skills of children with ASD.

Thus, this training is a fun and motivating method of targeting physical condition challenges in individuals with ASD. Since the goal of motivating children to exercise was achieved simply and easily as well as the training, this method can be also applied at home and it is encouraged for parents.

While the current study had a number of strengths, key limitations exist. One limitation is that this study was not designed to understand the mechanisms underlying poorer postural stability in ASD. Nevertheless, these results answer key questions regarding how postural stability may be impacted in ASD on unstable surfaces and who is likely to be the most impacted. Importantly, these findings can speak to clinical decision making when trying to treat balance challenges in ASD and may lay the groundwork for future research to examine the underlying mechanisms. Similarly, this study was not designed to examine fine or gross motor skills that may interact with postural stability impairments in ASD. Future research is needed to examine postural stability in light of other potential motor challenges, and longitudinal studies will be needed to determine whether the development of postural stability is similar in ASD compared to typical development.

## 5. Conclusions

The results of the study showed that applying a specific exercise programme in the gym and on the smart board and using it consistently for 5 weeks improved the physical condition of children with ASD. The simple exercise programme used in the gym and the additional exercises on the smart board resulted in a positive change in children’s balance, coordination, and motor skills. The resulting mean change, calculated from the scores of each group that attended the sessions, shows that the group that received physical therapy sessions on a smart board had the greatest change of 1.58 points. This shows that the opportunity to play virtual reality games not only motivates children with ASD but also encourages them to work harder. On the other hand, it is a simple and easy way to engage children in different types of physical exercise. A slightly smaller change of 1.51 was seen in the group that received gym lessons. Naturally, the constant practice of different physical exercises in the gym improves the physical condition. However, it should be noted that working in the gym was more psychologically challenging for the children with ASD due to their lack of desire and motivation to do the exercises. The control group shows the smallest change of 0.35 points. Thus, there is no significant difference between the changes of the group working on the smart board and the group working in the gym, but the changes of both groups are significantly different from the control group: about 1.2 points. The performed study has shown that the simple exercise programmes gave good results on children’s balance, coordination and motor skills. Both physical therapy methods are relatively simple and easy to apply at home; therefore, parents can make a significant contribution to improving children’s physical condition.

## Figures and Tables

**Table 1:** The kinesiotherapy programs.

Rub your feet on the massage machine	1 min.
Stable ball grip	1.min.
Lying on your back, on a large roller, stretching	3 min.
Steps by foot placed on the floor	4 repetitions
Steps by foot placed on the floor turned sideways	4 repetitions
Jump over three obstacles with both feet	8 repetitions
Throwing a ball, first with a small ball, then a bigger one, if you are doing well, then we will throw with one hand alternately	4 repetitions, 10 passes each
Jumping by colour with two legs and one in five coloured arcs placed on the ground	8 jumps, 2 series
Shuttle running	1 min., 2 series
Standing on an unstable plate for	1 min.
Climbing the soft stairs and walking through the bench	4 repetitions
Lie on your stomach, on a physiotherapy ball, roll forward with the help of your hands and go back to lay your knees on the ground	2 min.
Raising the medical ball above the head and lowering it to the abdomen	5 repetitions, 2 series
Opposite arm and leg reach	6 repetitions, 2 series
Breathing - Inhale through your nose with your hands rising, falling your hands exhale through your mouth.	5 repetitions

**Table 2:** Pre- and post-test results of balance, coordination and motor skills of children with ASD who participated in physiotherapy sessions and a control group.

	Smart Board Group		Gym Group		Control Group	
Test	Before KIN	After KIN	Before KIN	After KIN	Before KIN	After KIN
The Modified Berg functional balance scale	38.3 ± 6.4	41.3 * ± 5.9	36.9 ± 10.1	41.5 * ± 8.8	38.9 ± 8.7	39.7 * ± 8.5
The non-equilibrium coordination patterns	33.5 ± 10.7	38.5 * ± 9.9	35.9 ± 11.6	39.4 * ± 11	36.3 ± 9.4	37.7 * ± 9.2
BOTMP						
Gross motor						
Running speed and agility	0.5 ± 0.7	0.5 ± 0.7	0.5 ± 0.7	1 * ± 0.9	0.6 ± 0.7	0.6 ± 0.7
Balance	16.4 ± 4.6	17.8 * ± 5	14.9 ± 7.9	16 * ± 7.8	13.3 ± 5.7	13.6 ± 6
Bilateral coordination	4 ± 2.3	5.4 * ± 2.3	4.8 ± 3	5.6 * ± 3.2	4 ± 3.5	4.1 ± 3.5
Strength	12.3 ± 3.7	12.5 ± 3.9	12.1 ± 5.9	13.7 * ± 6	11.1 ± 5	11.3 ± 5.1
General motor						
Upper body coordination	8.2 ± 4.3	9.8 * ± 4.8	8.2 ± 4	9 * ± 4.4	7.8 ± 4.6	8 ± 4.6
Fine motor						
Reaction speed	5.1 ± 2.4	5.6 * ± 2.6	5.2 ± 2.6	6.1 * ± 2.5	4.7 ± 2.3	4.9 ± 2.5
Visual motor control	11.2 ± 3.3	12.8 * ± 3.4	10.8 ± 5.3	11.1 ± 5	8.5 ± 5.6	8.7 ± 5.4
Speed and dexterity of the upper body	21.7 ± 10.3	22.8 * ± 10.1	23.1 ± 12.4	24.1 * ± 12.7	20.2 ± 13.2	20.3 ± 13.1

Note: * *p* < 0.05 statistically significant result after physiotherapy. ± standard deviation.

## Data Availability

The data presented in this study are available on request from the corresponding author. The data are not publicly available due to privacy reasons.
